# The EZH2 selective inhibitor ZLD1039 attenuates UUO-induced renal fibrosis by suppressing YAP activation

**DOI:** 10.1186/s43556-025-00276-5

**Published:** 2025-06-06

**Authors:** Qingling Xia, Fujiang Xu, Lidan Zhang, Wenfei Ding, Jiang Liu, Jing Liu, Minhao Chen, Santao Ou, Yong Xu, Li Wen

**Affiliations:** 1 Department of Nephrology, Sichuan Clinical Research Center for Nephropathy and Metabolic Vascular Diseases Key Laboratory of Sichuan Province, The Affiliated Hospital of Southwest Medical University, Luzhou, China; 2 Department of Oncology, The Affiliated Hospital of Southwest Medical University, Luzhou, China; 3 Laboratory of Anesthesia & Critical Care Medicine, Translational Neuroscience Center, West China Hospital of Sichuan University, Chengdu, China; 4 Department of Urology, The Affiliated Hospital of Southwest Medical University, Luzhou, China; 5 Clinical Medical College, Southwest Medical University, Luzhou, China; 6 Department of Endocrinology and Metabolism, The Affiliated Hospital of Southwest Medical University, Luzhou, China

**Keywords:** Enhancer of zeste homolog 2, Large tumor suppressor 1, Yes-associated protein 1, Inflammation, Renal fibrosis

## Abstract

Renal fibrosis is a manifestation of the progression of chronic kidney disease (CKD) and chronic inflammation is a main driver in the development of renal fibrosis. Yes-associated protein (YAP), acting as a transcriptional co-activator within the Hippo signaling pathway, has been implicated in renal fibrosis. Enhancer of zeste homolog 2 (EZH2) exhibits high expression level in renal fibrosis induced by unilateral ureteral obstruction (UUO), yet the interplay between YAP and EZH2 in renal fibrosis remains to be elucidated. ZLD1039, a selective inhibitor of EZH2, has demonstrated protective effects against cancer and acute kidney injury (AKI). In this study, we conducted a systemic pharmacological investigation to determine if ZLD1039 treatment mitigates UUO-induced renal inflammation and fibrosis through modulation of the Hippo-YAP pathway. Our results revealed that UUO triggered renal inflammation and collagen deposition, with significant activation of YAP. Notably, ZLD1039 treatment effectively alleviated renal inflammation and fibrosis, while inhibiting the expression and nuclear translocation of YAP. Mechanically, we observed a notable down-regulation of large tumor suppressor homolog 1 (LATS1) in parallel with the up-regulation of EZH2. Furthermore, inhibition of EZH2 by ZLD1039 was linked to the up-regulation of LATS1 expression and YAP inactivation. Similarly, in vitro pharmacological inhibition of EZH2 by ZLD1039 resulted in elevated LATS1 expression and diminished YAP activation. Collectively, our findings suggest that ZLD1039, a selective inhibitor of EZH2, likely attenuates renal inflammation and fibrosis probably by up-regulating LATS1 and inhibiting YAP activation. This mechanistic link between EZH2 and YAP provides a fresh perspective on treating renal fibrosis.

**Supplementary Information:**

The online version contains supplementary material available at 10.1186/s43556-025-00276-5.

## Introduction

Chronic kidney disease (CKD) affects approximately 10% of the global population, with a steady increase in both incidence and mortality, presenting a significant health challenge [1, 2]. Renal fibrosis, a hallmark pathological feature of CKD, is characterized by the proliferation of myofibroblasts and fibroblasts, the epithelial-to-mesenchymal transition (EMT) of renal cells, and the excessive accumulation of extracellular matrix (ECM) in renal tissues [3, 4]. Ureteral obstruction, a common cause of CKD, often leads to renal fibrosis [5]. Commonly, various pathophysiological processes contribute to obstructive nephropathy, including mechanical stress, cellular migration and infiltration, inflammation, myofibroblast accumulation, increased ECM deposition, and tubulointerstitial fibrosis [6]. Inflammation is currently recognized as the primary driver of renal fibrosis, with the mechanism of unilateral ureteral obstruction (UUO)-induced renal fibrosis involving the transition of macrophages to myofibroblasts [7]. However, the underlying molecular mechanisms behind the inflammatory responses in renal fibrosis remain elusive, and effective treatments to delay the progression of obstructive nephropathy are lacking. Hence, there is an urgent need to elucidate the inflammatory mechanism and develop novel therapeutic agents to protect against renal fibrosis in CKD.

The Hippo signaling pathway, composed of several core proteins including mammalian sterile 20-like 1/2 (MST1/2), salvador (SAV1), MOB kinase activator 1 (MOB1), large tumor suppressor homolog 1/2 (LATS1/2), yes-associated protein (YAP) and transcriptional co-activator with PDZ-binding motif (TAZ) [8, 9]. The transcriptional effectors YAP and TAZ are crucial members of the Hippo signaling pathway, and plays a crucial role in regulating cell proliferation, tissue regeneration, epithelial homeostasis, cell survival, and immune modulation [9, 10]. Under normal conditions, MST1/2 forms a complex with SAV1 to phosphorylates LATS1/2, which subsequently phosphorylates YAP and TAZ, targeting YAP for proteasomal degradation and inhibiting its transcription activity, thereby activating the Hippo signaling pathway. Conversely, when YAP phosphorylation is blocked, YAP translocates to the nucleus and binds to TEA domains (TEAD), acting as a transcriptional co-activator to regulate gene expression, thereby inhibiting the Hippo signaling pathway. Notably, the Hippo signaling pathway has been implicated in organ development, cancer, and obesity-induced adipose tissue fibrosis [9]. Its role in kidney diseases was first identified in cystic kidney disease [11], and subsequent studies have highlighted its involvement in various kidney diseases. For instance, *Zheng *et al. [12] reported that Hippo-YAP signaling mediates tubular maladaptive repair by upregulating MCP-1 and promoting inflammation in ischemic acute kidney injury (AKI). However, the upstream mechanisms of YAP remain poorly understood.

Epigenetics, defined by heritable changes in gene expression or cell phenotype without altering the DNA sequence, encompasses DNA methylation, RNA interference and histone modification [13]. Enhancer of zeste homolog 2 (EZH2), a histone methyltransferase and a key component of the polycomb repressive complex 2 (PRC2), catalyzes the trimethylation of histone H3 at lysine 27 (H3K27me3) by transferring a methyl group from S-adenosylmethionine (SAM) to H3 K27, thereby silencing target genes [14]. ZLD1039, a selective EZH2 inhibitor, competes with SAM to inhibit EZH2 activity [15]. Increasing evidence suggests that EZH2 plays a pivotal role in renal fibrosis by regulating EMT and activating M2 macrophage polarization [16, 17]. Additionally, EZH2 has been shown to regulate the Hippo signaling pathway in esophageal squamous cell carcinoma and breast cancer [18, 19]. Our previously study demonstrated that EZH2-mediated inflammation contributes to AKI, and that inhibiting EZH2 with ZLD1039 in renal tubular cells effectively mitigates renal inflammation and ameliorates kidney injury [20]. However, the potential of ZLD1039 as a protective agent against renal fibrosis remains largely unexplored. Moreover, the precise mechanisms through which EZH2 regulates renal inflammation and fibrosis are not yet fully elucidated. Given that, we aim to investigate the potential interplay between EZH2 and the Hippo signaling pathway in renal inflammation and fibrosis, hypothesizing that inhibiting EZH2 with ZLD1039 could confer therapeutic benefits in renal inflammation and fibrosis by suppressing YAP activation. This mechanistic investigation could provide new insights into the pathogenesis of renal fibrosis. Moreover, the clinical value of this research is substantial. Renal fibrosis is a major contributor to CKD progression, and current treatments are limited. ZLD1039 may offer a new therapeutic strategy to mitigate renal fibrosis. The potential to suppress YAP activation through EZH2 inhibition could provide significant benefits in reducing renal inflammation and fibrosis, ultimately improving patient outcomes.

In this study, we established a renal fibrosis model via UUO and subsequently evaluated the expression level of EZH2, proteins associated with the Hippo signaling pathway, inflammatory cytokines, and fibrosis maker proteins in UUO kidney tissues. Thereafter, ZLD1039 was administered to UUO rats for a defined period, and its protective effects against renal inflammation and fibrosis were assessed. Additionally, the correlation between EZH2 and the Hippo signaling pathway-related proteins was examined following ZLD1039 treatment.

## Results

### ZLD1039 inhibits UUO-induced renal fibrosis via EZH2/H3K27me3-dependent pathway

**Fig. 1:**
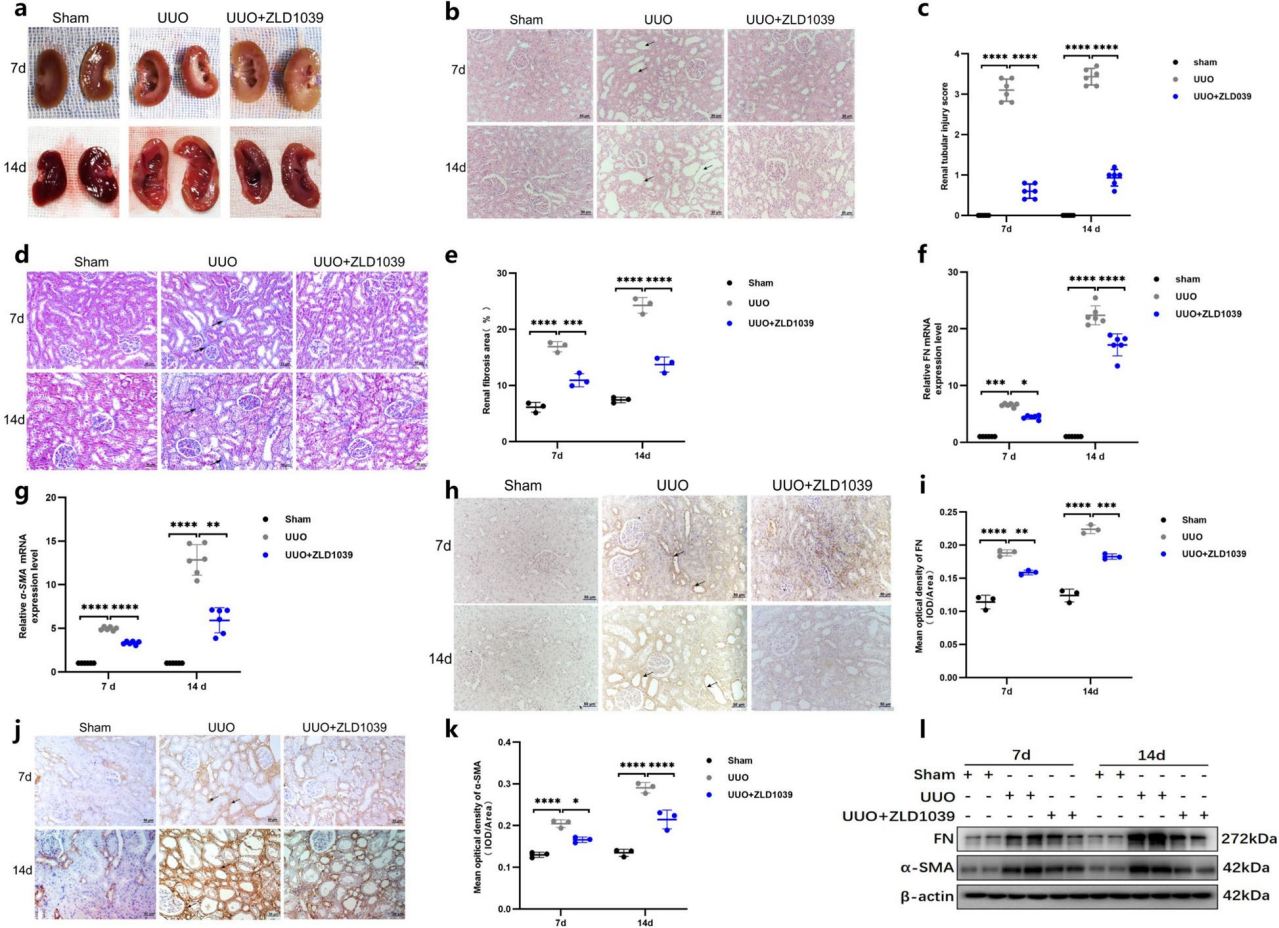
ZLD1039 inhibits UUO-induced renal fibrosis. **a** The appearance of kidney tissues following unilateral ureteral ligation; **b**, **c** Representative sections of H&E staining of kidney tissues (original magnification × 200) and renal tubular injury scores were calculated according to the criterion in materials and methods; Data are represented as means ± SDs (*n* = 6); **d**, **e** Representative sections of Masson’s trichrome staining of kidney tissues (original magnification × 200) and renal fibrosis scores were calculated. Data are represented as means ± SDs (*n* = 3); **f**, **g** The mRNA levels of FN and α-SMA; Data are represented as means ± SDs (*n* = 6); **h**, **i** Representative sections of IHC staining of FN (original magnification × 200) and mean optical density of FN; **j**, **k** Representative sections of IHC staining of α-SMA (original magnification × 200) and mean optical density of α-SMA; Data are represented as means ± SDs (*n* = 3); **l** The protein expression levels of FN and α-SMA were quantified by normalized with β-actin. * *p* < 0.05; ** *p* < 0.01; *** *p* < 0.001; **** *p* < 0.0001. All western blotting analyses were performed in two randomized mice from each group, and the experiments were repeated in triplicate

ZLD1039 has been reported as a selective inhibitor of EZH2 with anti-tumor effects in breast cancer and renal protective effects [20, 21]. The chemical structural formula of ZLD1039 is shown in previous research [20]. Our previous studies demonstrated that ZLD1039 is no-toxic effects to the heart, liver, spleen, and kidneys, thereby confirming its safety [20]. To investigate the effect of ZLD1039 on UUO-induced renal fibrosis in rats, HE and Masson’s trichrome staining were used to evaluate renal tubular injury and fibrosis at different stages of renal fibrosis development. As shown in Fig. 1a, kidney tissues in the UUO group exhibited varying degrees of renal calyceal and pelvic dilation, which worsened with prolonged obstruction. However, ZLD1039 treatment significantly improved this dilation. HE staining showed that ZLD1039 treatment attenuated tubular dilation, loss of brush border, epithelial desquamation, and inflammatory cell infiltration in UUO-induced kidneys (Fig. 1b-c). Masson’s trichrome staining revealed that collagen deposition was significantly increased in UUO-induced kidneys, whereas ZLD1039 treatment markedly reduced collagen accumulation (Fig. 1d-e). Moreover, the mRNA levels of fibronectin (FN) and α-smooth muscle actin (α-SMA) were obviously lower in kidneys from the ZLD1039-treated rats compared to those in UUO-induced rats (Fig. 1f-g). Besides, immunohisto-chemistry (IHC) staining for FN (Fig. 1h-i) and α-SMA (Fig. 1j-k) also demonstrated a significant reduction in these proteins in ZLD1039-treated rats compared with UUO-induced rats. Consistent with these findings, western blotting showed that renal FN and α-SMA proteins levels were decreased in ZLD1039-treated rats compared with UUO-induced rats (Fig. 1l). Hence, these data suggest that ZLD1039 may effectively attenuate the progression of UUO-induced renal fibrosis.

**Fig.2:**
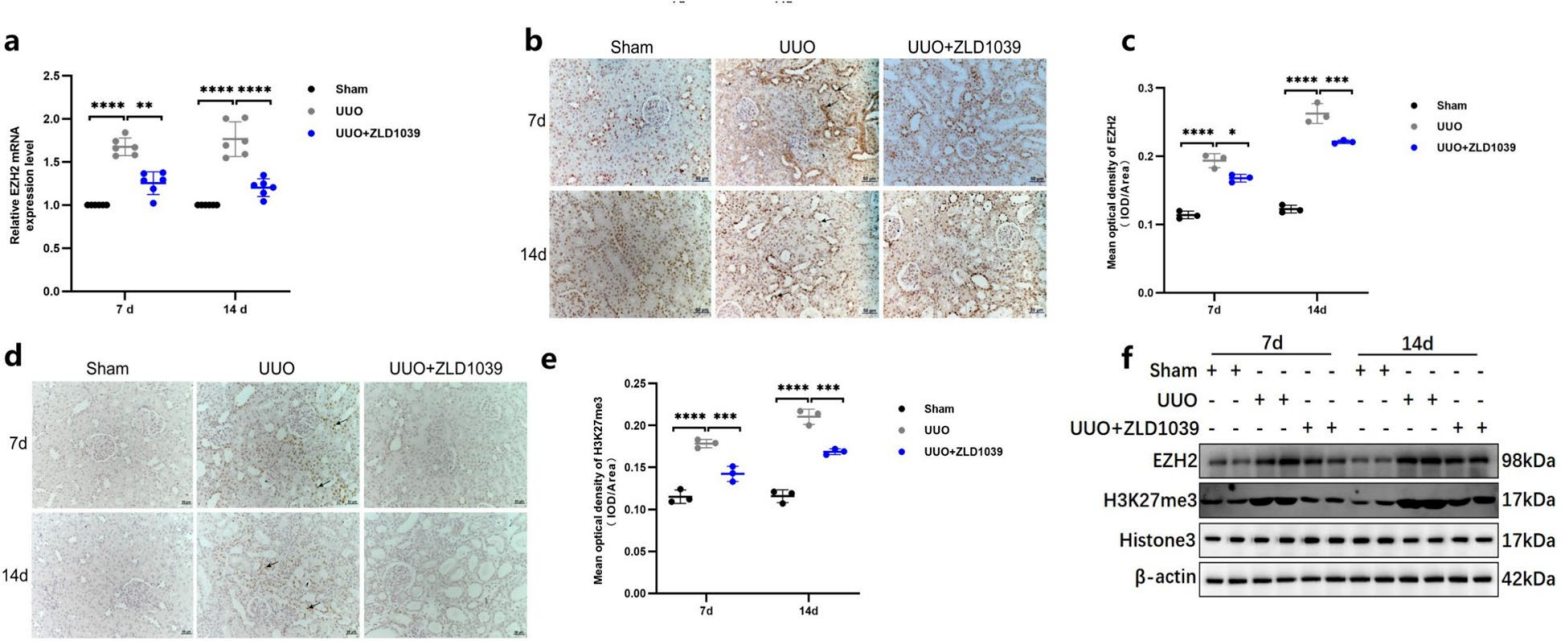
ZLD1039 indeed suppresses EZH2 and H3K27me3 expression in UUO rats. **a** The mRNA level of EZH2; **b**-**e** Photomicrographs (original magnification × 200) illustrates EZH2 and H3K27me3 IHC staining of kidney tissues from sham or UUO mice with/without ZLD1039 administration and the mean optical density of EZH2 and H3K27me3; Data are represented as means ± SDs (*n* = 3); **f** The protein expression levels of EZH2 and H3K27me3 were quantified by normalized with β-actin and Histone H3; * *p* < 0.05; ** *p* < 0.01; *** *p* < 0.001; **** *p* < 0.0001. All western blotting analyses were performed in two randomized mice from each group, and the experiments were repeated in triplicate

*Song *et al*.* [21] have shown that ZLD1039 selectively inhibits EZH2, with minimal effects on other histone methyltransferases, including EZH1, SUV39H1, G9a, SETD7, SUV39H2, SMYD2, PRDM9, and SETD8. To determine whether the anti-fibrotic effect of ZLD1039 is mediated by EZH2 inhibition, we examined the mRNA and protein expression levels of EZH2 in the kidneys of rats with UUO-induced renal fibrosis. As show in Fig. 2, the expression levels of EZH2 (Fig. 2a-c, f) and H3K27me3 (Fig. 2 d-f) were significantly elevated in the kidneys of UUO-induced rats compared with the sham group. In contrast, ZLD1039 treatment markedly reduced the expression of both EZH2 and H3K27me3 compared with the UUO group. Collectively, these results indicate that the anti-fibrotic effect of ZLD1039 is mediated by the inhibition of EZH2 expression and activity.

### ZLD1039 treatment attenuates UUO-induced renal inflammation

**Fig.3:**
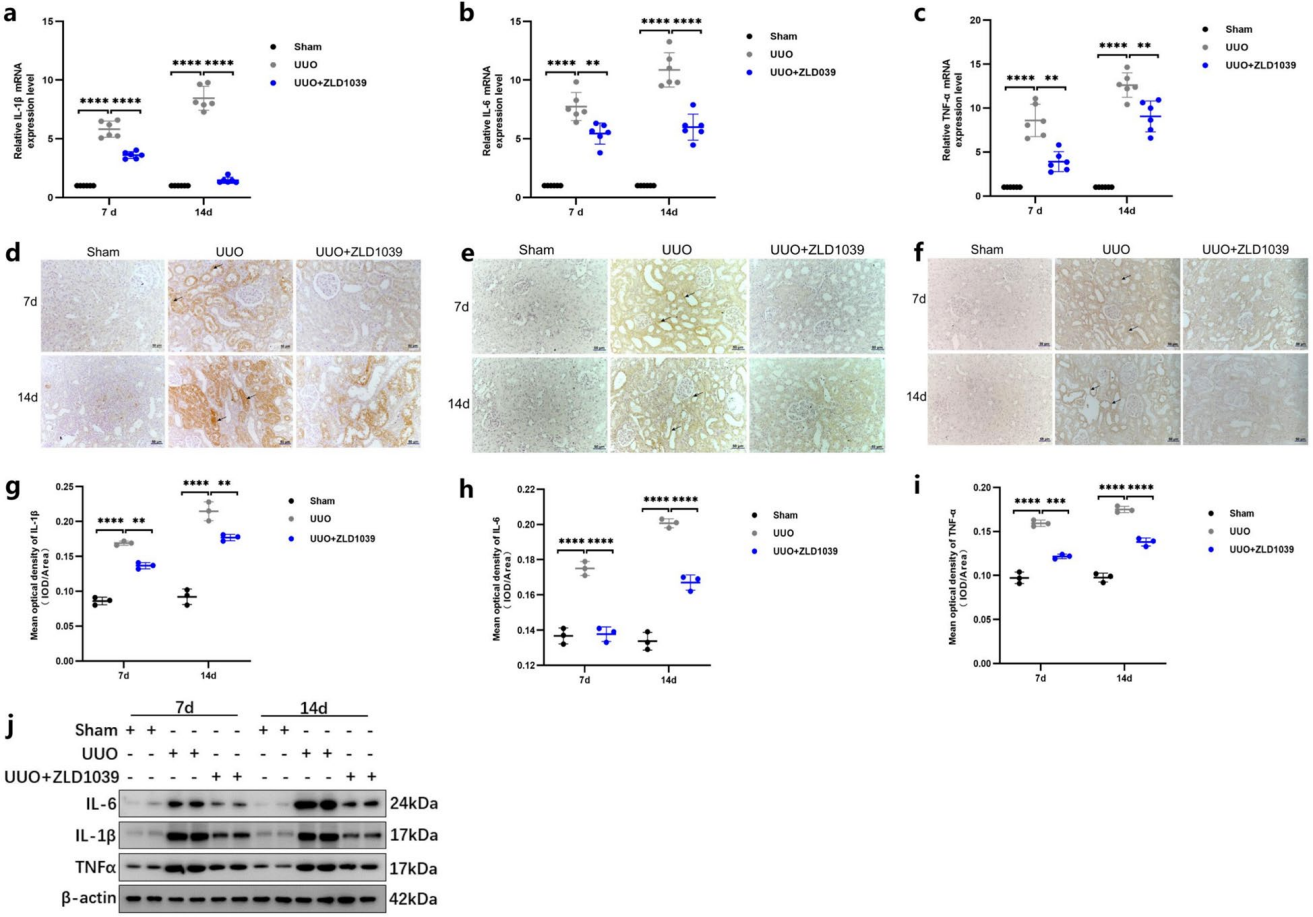
EZH2 inhibition by ZLD1039 attenuates renal inflammation in UUO rats. **a**-**c** The mRNA levels of IL-1β, IL-6 and TNFα; Data are represented as means ± SDs (*n* = 6); **d**, **g** Representative sections of IHC staining of IL-1β (original magnification × 200) and the mean optical density of IL-1β; **e**, **h** Representative sections of IHC staining of IL-6 (original magnification × 200) and the mean optical density of IL-6; **f**, **i** Representative sections of IHC staining of TNFα (original magnification × 200) and the mean optical density of TNFα; Data are represented as means ± SDs (*n* = 3); **j** The protein expression levels of IL-1β, IL-6 and TNFα were quantified by normalized with β-actin; ** *p* < 0.01; *** *p* < 0.001; **** *p* < 0.0001. All western blotting analyses were performed in two randomized mice from each group, and the experiments were repeated in triplicate

To further elucidate the role of ZLD1039 in UUO-induced renal inflammation, we investigated its effects on the expression of key inflammatory cytokines, including IL-1β, IL-6 and TNFα. UUO surgery significantly increased the mRNA levels of IL-1β, IL-6 and TNFα (Fig. 3a-c), with the increase becoming more pronounced with longer obstruction duration. However, ZLD1039 treatment significantly reduced the mRNA levels of IL-1β, IL-6 and TNFα (Fig. 3a-c). Similarly, the protein levels of these inflammatory cytokines were elevated in the UUO group compared with the sham group, but were markedly decreased in the ZLD1039-treated group (Fig. 3d-j). These data collectively demonstrate that ZLD1039 significantly suppresses UUO-induced renal inflammation by downregulating the expression of key inflammatory cytokines.

### Blockade of EZH2 increases the expression and activation of LATS1 in UUO-induced renal fibrosis

**Fig.4:**
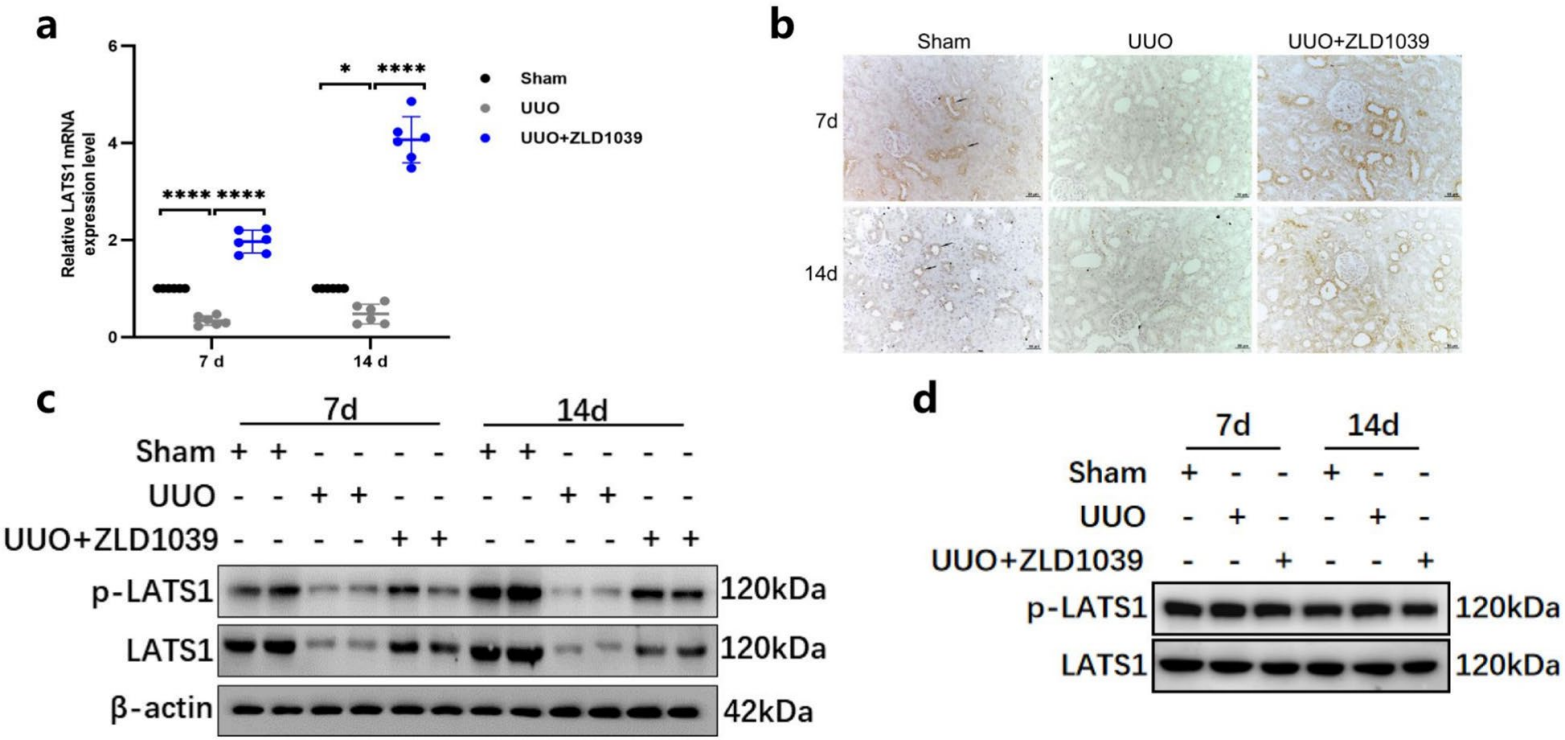
EZH2 inhibition by ZLD1039 increases LATS1 expression in UUO rats. **a** The mRNA level of LATS1; Data are represented as means ± SDs (*n* = 6); **b** Representative sections of IHC staining of p-LATS1 (original magnification × 200). **c** The protein expression level of LATS1 and p-LATS1 were quantified by normalized with β-actin; **d** The protein expression level of p-LATS1 were quantified by normalized with LATS1; * *p* < 0.05; **** *p* < 0.0001. All western blotting analyses were performed in two randomized mice from each group, and the experiments were repeated in triplicate

We previously discovered that inhibiting EZH2 with ZLD1039 can exert anti-inflammatory and anti-fibrotic effects in the kidney, although the precise mechanisms remain unknown. Given that EZH2 plays a crucial role in renal cell carcinoma by directly binding to the LATS1 promoter to inhibit its expression and promote tumor growth [22], we hypothesized that the protective effects of ZLD1039 in UUO-induced renal fibrosis might be mediated by LATS1 expression and activation. Not surprisingly, the mRNA expression level of LATS1 was dramatically decreased in rats with UUO-induced renal fibrosis (Fig. 4a). Nevertheless, administration of ZLD1039 led to a notable increase in LATS1 expression (Fig. 4a). IHC revealed strong p-LATS1 staining in the cytoplasm of renal tubular cells in the sham-operated rats at both 7 and 14 days, whereas UUO surgery significantly reduced p-LATS1 expression (Fig. 4b). In contrast, ZLD1039 treatment restored p-LATS1 expression, indicating that ZLD1039 promotes LATS1 activation (Fig. 4b). Western blotting also showed that total LATS1 and phosphorylated LATS1 (p-LATS1) protein levels were significantly lower in the UUO group compared with those in the sham group (Fig. 4c). ZLD1039 treatment recovered total LATS1 and p-LATS1 levels by inhibiting EZH2 expression and activity (Fig. 4c), suggesting that LATS1 is indeed a crucial target of EZH2. To intuitively observe the changes in LATS1 and p-LATS1, we normalized LATS1 before analysis. The results demonstrated that the changes in p-LATS1 protein levels were synchronous with those of LATS1 after normalization (Fig. 4d). This observation together with the results shown in Fig. 4c, suggests that alterations in the mRNA and protein levels of LATS1 may exert their effects by influencing p-LATS1 levels. Given that EZH2 primarily exerts transcriptional repression through H3K27me3, we further analyzed the overlap between H3K27me3 and the LATS1 promoter region using the Cistrome Data Browser. The ChIP-seq results showed a distinct enrichment peak of H3K27me3 in the promoter region of LATS1 (Supplementary Material. Fig. S1), suggesting that EZH2 may directly inhibits LATS1 transcription via H3K27me3. However, the specific mechanisms underlying this interaction warrant further investigation.

### EZH2 inhibition may suppress YAP activation in rats with UUO-induced renal fibrosis

**Fig.5:**
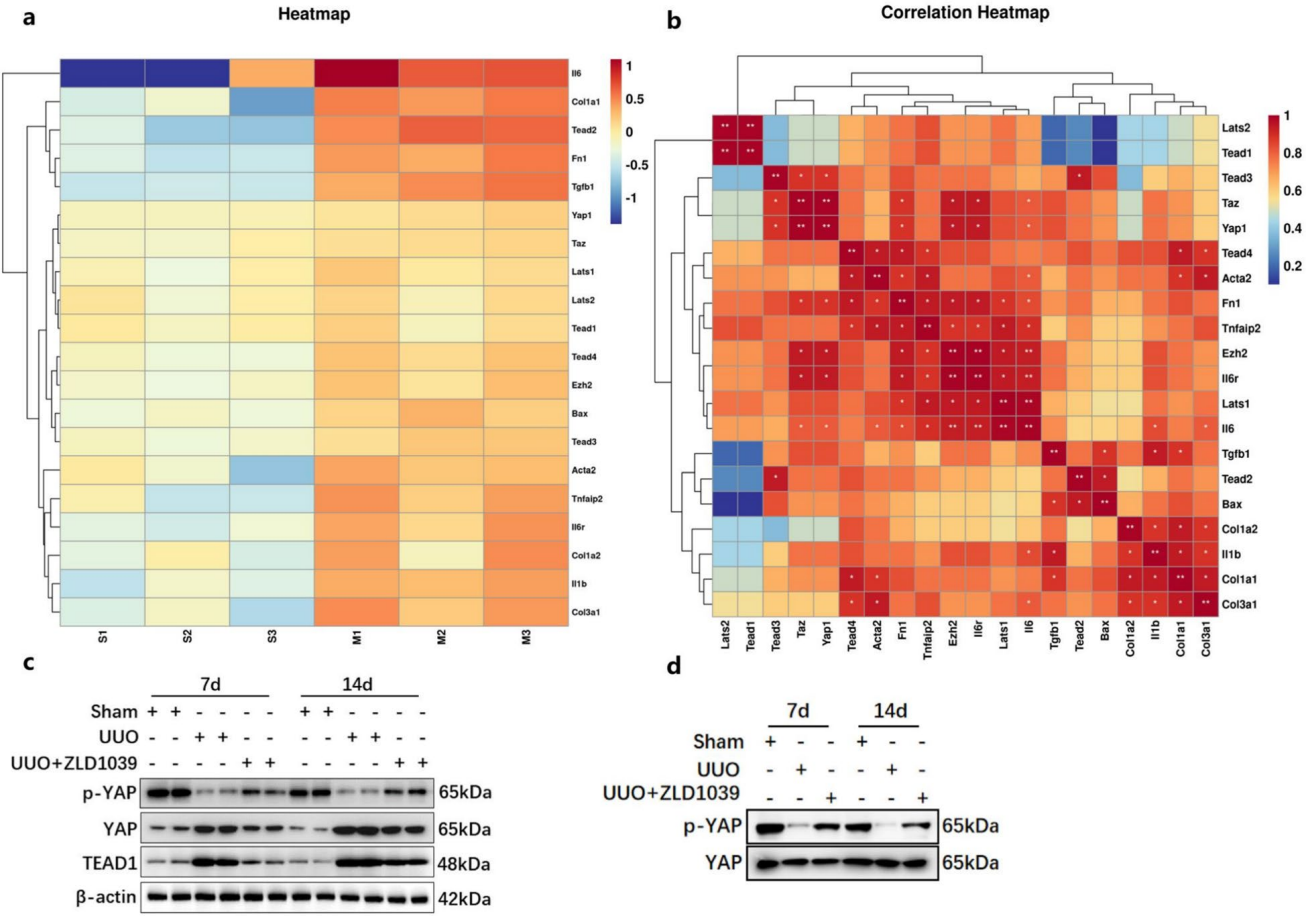
EZH2 inhibition by ZLD1039 blocks YAP activation in UUO rats. **a**, **b** The cluster heatmap and correlation heatmap were conducted to present differentially expressed genes in the GSE181380 dataset (*n* = 3). **c** The protein expression levels of YAP, p-YAP and TEAD1 were quantified by normalized with β-actin; **d** The protein expression level of p-YAP were quantified by normalized with YAP; All western blotting analyses were performed in two randomized mice from each group, and the experiments were repeated in triplicate

To determine the signaling pathways involved in the anti-fibrotic effect of ZLD1039, we focused on the Hippo signaling pathway, particularly due to the pivotal role of LATS1 as a key tumor suppressor within this pathway. Specifically, RNA-sequencing data from the Gene Expression Omnibus database under accession number GSE181380 revealed that the expression levels of both EZH2 and YAP were simultaneously up-regulated in the kidneys of UUO rats at 14 days, with a significant positive correlation observed between these two proteins (Fig. 5a, b). Western blotting revealed that YAP and TEAD1 (Fig. 5c) expression was significantly elevated in UUO-injured rats, accompanied by decreased level of phosphorylated YAP (p-YAP) (Fig. 5c). In contrast, ZLD1039 administration reduced YAP and TEAD1 protein levels while increased p-YAP expression (Fig. 5c). Additionally, we used YAP as a reference for normalization. The results showed that the expression of p-YAP in the model group also decreased, while ZLD1039 increased the expression of p-YAP (Fig. 5d). These findings collectively suggest that YAP is a crucial mediator of EZH2-driven UUO-induced renal fibrosis, and its inhibition by ZLD1039 contributes to the anti-fibrotic effect.

### High LATS1 and low EZH2 expression are associated with ZLD1039 anti-fibrotic effect in TGFβ-induced renal TECs

**Fig.6:**
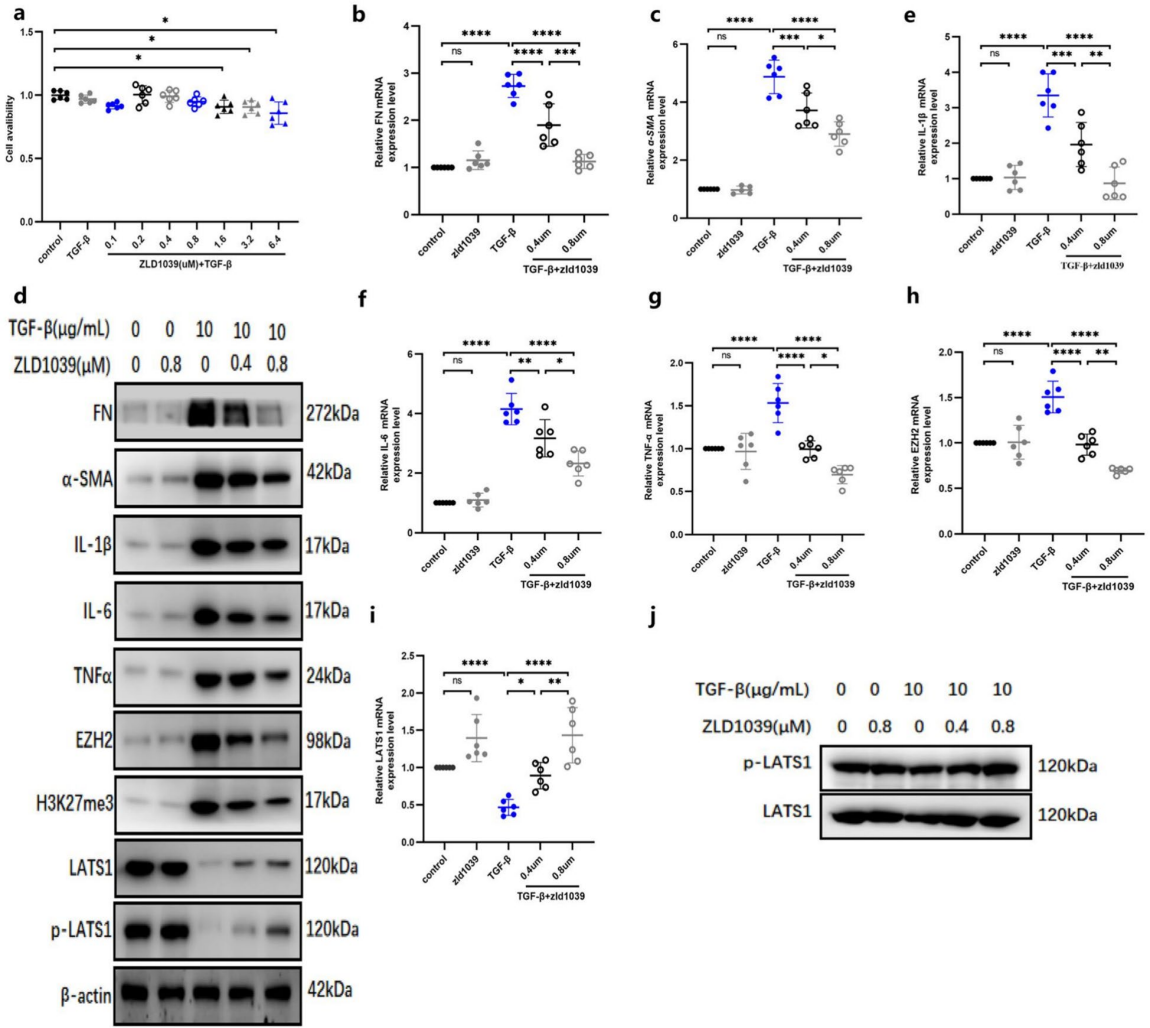
ZLD1039 alleviates TGFβ-induced renal TECs inflammation and fibrosis by inhibiting EZH2 and elevating the expression of LATS1. **a** The cell viability of TECs under the stimulation of TGFβ and ZLD1039. Data are represented as means ± SDs (*n* = 6); **b**-**e**, **f**-**i** The mRNA levels of FN, α-SMA, IL-1β, IL-6, TNFα, EZH2 and LATS1; Data are represented as means ± SDs (*n* = 6); **d** The protein expression levels of FN, α-SMA, IL-1β, IL-6, TNFα, EZH2, H3K27me3, LATS1 and p-LATS1 were quantified by normalized with β-actin; **j** The protein expression level of p-LATS1 were quantified by normalized with LATS1 in TGFβ-induced TECs; * *p* < 0.05; ** *p* < 0.01; *** *p* < 0.001; **** *p* < 0.0001; ^ns^*p* > 0.05. All Western Blotting analyses were repeated in triplicate

To elucidate the role of EZH2 and LATS1 in the anti-fibrotic effect of ZLD1039 in proximal tubular epithelial cells (TECs), we induced fibrosis in TECs using TGFβ and treated the cells with ZLD1039. We initially employed the CCK-8 assays to assess the cytotoxicity of various concentrations of ZLD1039 in NRK-52E cells. The results revealed that ZLD1039 was cytotoxic at concentrations above 1.6μΜ (Fig. 6a). Therefore, we chose concentrations of 0.4 and 0.8 μM for further experiments. After TGFβ stimulation, the mRNA and protein levels of FN and α-SMA were significantly up-regulated, but these effects were attenuated by ZLD1039 treatment (Fig. 6b-d). Additionally, ZLD1039 inhibited the up-regulation of pro-inflammatory cytokines IL-1β, IL-6 and TNFα induced by TGFβ in TECs (Fig. 6d-g). Moreover, TGFβ significantly increased the expression of EZH2 (Fig. 6d, h) while reducing the expression of LATS1 and p-LATS1 (Fig. 6d, i). However, ZLD1039 treatment reversed these changes, suggesting that its anti-fibrotic effect is associated with the LATS1 up-regulation. In TGFβ stimulated cells,we observed that using LATS as a reference, the level of p-LATS fluctuated in accordance with the changes in LATS expression (Fig. 6j). Collectively, these findings demonstrate that LATS1 is a key target of EZH2 in TECs fibrosis.

### ZLD1039 treatment blocks YAP activation in TECs exposed to TGFβ

**Fig.7:**
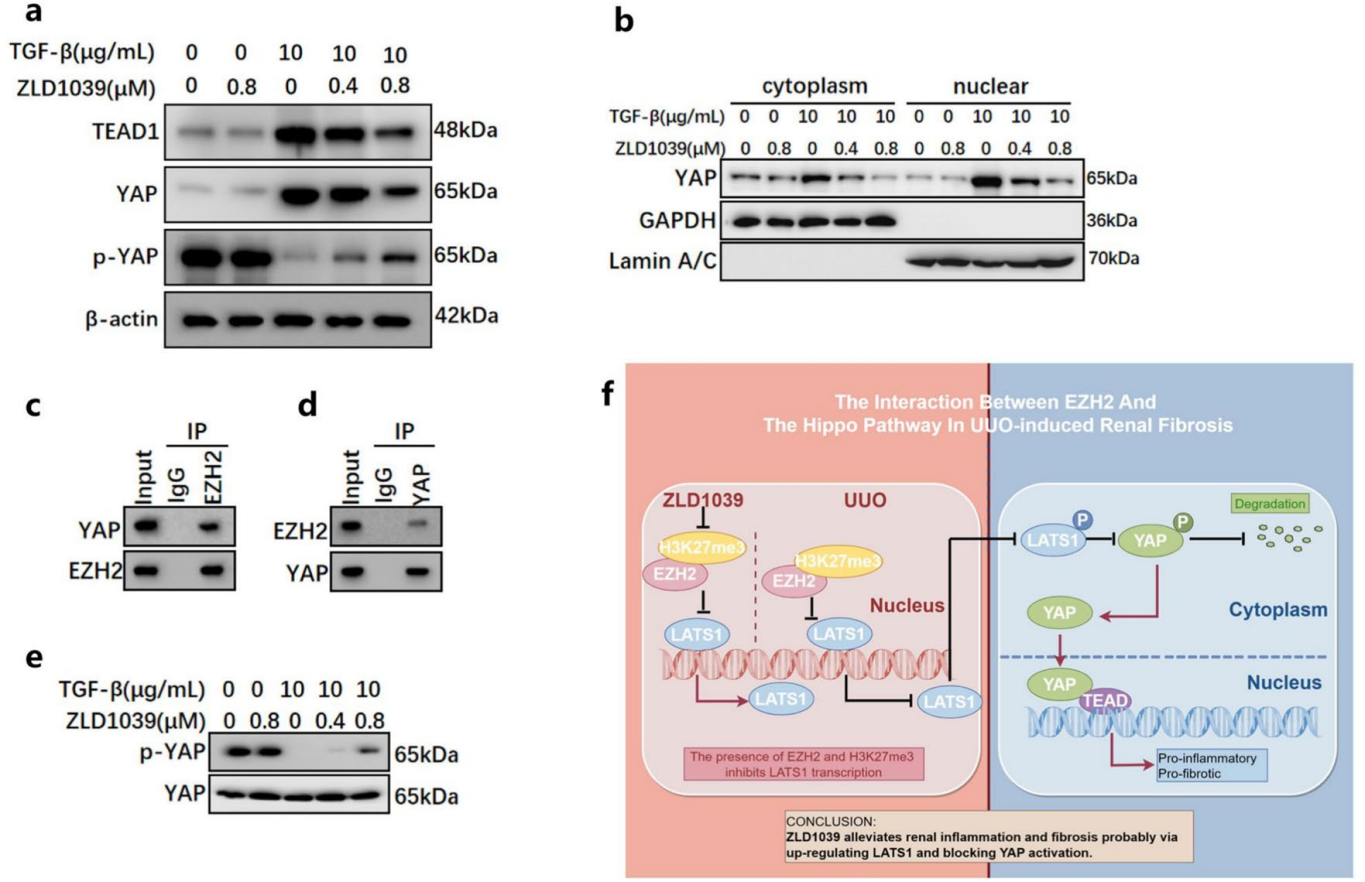
ZLD1039 treatment represses YAP activation in TGFβ-induced TECs. **a** The protein expression levels of YAP, p-YAP and TEAD1 were quantified by densitometry and normalized with β-actin; **b** The expression levels of YAP in cytoplasm and nucleus; **c**,**d** The interactions between EZH2 and YAP were detected by Co-Immunoprecipitation (Co-IP) in NRK-52E cells. **e** The protein expression level of p-YAP was quantified by normalized with YAP in TGFβ-induced TECs; **f** The interaction between EZH2 and the Hippo pathway in UUO-induced renal fibrosis (This picture is drawn using Figdraw). All Western Blotting analyses were repeated in triplicate

To investigate YAP activation in response to TGFβ stimulation and the effects of ZLD1039, TECs were treated with TGFβ (10 ng/ml) to induce fibrosis. TGFβ stimulation led to increased levels of YAP and TEAD1, while reducing p-YAP level (Fig. 7a). To further investigate the nuclear localization of YAP, we performed nuclear-cytoplasmic separation on cells stimulated with TGFβ. The results showed that TGFβ upregulated YAP protein levels in both the cytoplasm and nucleus, with a more pronounced increase in the nucleus (Fig. 7b), indicating that TGFβ promotes YAP nuclear translocation. In contrast, ZLD1039 treatment increased p-YAP level and dose-dependent decreased YAP and TEAD1 levels (Fig .7a). This was associated with YAP degradation and activation of the Hippo signaling pathway. Taken together, these findings suggest that ZLD1039 attenuates TGFβ-induced fibrosis in TECs by inhibiting YAP activation. Moreover, endogenous co-immunoprecipitation (Co-IP) assays were performed using anti-EZH2 or anti-YAP antibodies in lysates from NRK-52E cells. The results showed that YAP was detected in EZH2 immunoprecipitation (Fig. 7c), and EZH2 was detected in YAP immunoprecipitation (Fig. 7d), indicating an endogenous interaction between EZH2 and YAP. Further studies are needed to elucidate the specific molecular mechanisms and biological functions underlying this interaction. Similarly, in TGFβ stimulated cells, we used YAP as a reference for normalization and found that the expression of p-YAP also decreased in the TGFβ stimulated group, while ZLD1039 increased the expression of p-YAP (Fig. 7e), which is consistent with the results of Fig. 5d.

## Discussion

In recent years, the rapid advancement in the field of epigenetics, encompassing histone crotonylation, histone lactylation, and histone methylation, has ushered in a new era for the study of renal fibrosis. Inhibition of acyl-CoA synthetase short chain family member 2 (ACSS2) suppresses H3 K9 crotonylation-mediated IL-1β expression, thereby alleviating IL-1β-dependent macrophage activation and tubular cell senescence, and consequently delaying renal fibrosis [23]. This suggests that ACSS2 could serve as a potential therapeutic target for modulating H3 K9 crotonylation level and for attenuating renal fibrosis progression. In ischemia–reperfusion injury (IRI) mice, the elevated levels of the glycolytic enzyme 6-phosphofructo-2-kinase/fructose-2,6-biphosphatase 3 (PFKFB3) mediate increased H4 K12 lactylation. This, in turn, regulates the transcription and expression of genes in the NF-κB signaling pathway within tubular epithelial cells. Consequently, these changes promote renal inflammatory responses and the progression of renal fibrosis [24]. Additionally, our previous review summarized the roles of various methyltransferases and demethyltransferases in different renal diseases [25]. Furthermore, *Tsai *et al*.* recently reported that METTL3-mediated N6-methyladenosine (m6 A) mRNA modification can enhance the cGAS-STING signaling pathway, thereby promoting the progression of CKD [26]. Importantly, these studies have confirmed that epigenetic modifications play a crucial role in renal fibrosis, and the development of drugs targeting epigenetic regulatory enzymes or substrates may significantly delay or improve renal fibrosis. ZLD1039, a potent and selective inhibitor of EZH2, has demonstrated efficacy in preventing breast cancer and acute kidney disease [20, 21]. Our study elucidated the role of ZLD1039 in renal fibrosis and revealed that it exerts its anti-fibrotic effect by alleviating renal inflammation. Notably, our findings highlighted that LATS1 is a primary target of EZH2 and H3K27me3, and that YAP is activated in rats with UUO-induced renal injury. Importantly, ZLD1039 enhanced LATS1 transcription and up-regulated its phosphorylation level, subsequently increasing YAP phosphorylation, thereby ameliorating renal fibrosis. Collectively, our study suggests that ZLD1039 is a novel therapeutic agent for renal inflammation and fibrosis, with its specific molecular mechanism involving the up-regulation of LATS1 expression and the consequent inhibition of YAP activation.

Renal inflammation is recognized as a significant contributor to the progression of CKD [27, 28]. Notably, inflammatory processes and epigenetic modifications significantly impact the development and progression of kidney diseases [29, 30]. The histone methyltransferase EZH2 has been identified as a key factor in promoting renal tubular injury through mechanisms involving apoptosis and inflammatory responses [31]. Given the crucial role of EZH2 in promoting inflammatory processes, it is essential to identify the signaling pathway is involved in the EZH2-mediated inflammation. Previous studies have reported that EZH2 regulated intestinal inflammation via JNK signaling pathway [32]. Subsequently, the activation of HIF-1α transcription has been identified as a key mechanism underlying EZH2-induced inflammation and fibrosis [33]. Our previous research demonstrated that ZLD1039 alleviated cisplatin-induced AKI by up-regulating RKIP expression and blocking the NF-κB p65 pathway [20]. In the present study, we found that inhibiting EZH2 with ZLD1039 ameliorated renal inflammation and fibrosis by inactivating YAP, implying that epigenetics alterations may play a fundamental role in renal inflammation and fibrosis. It is noteworthy that the renal micro-inflammatory environment may exacerbate epigenetics changes, although this relationship remains underexplored. Thus, further investigation into the interplay between inflammation and epigenetics is warranted. Importantly, in mice with tubular MST1/2 deficiency, TNFα expression was significantly upregulated at 2 weeks, whereas YAP activation was not observed [34], indicating that MST deletion may induce renal inflammation through a pathway independent of YAP. Therefore, further investigation is needed to determine whether ZLD1039 ameliorates renal inflammation through non-YAP pathways.

The relationship between the Hippo signaling pathway and renal fibrosis has been extensively studied. Lan et al. [35] found that YAP activation promotes fibrosis in diabetic kidney disease (DKD). In mice with hypertensive renal disease, increased YAP and decreased p-YAP levels were associated with renal inflammation and fibrosis [36]. Our findings also indicate that in UUO-induced renal fibrosis, YAP expression is up-regulated while p-YAP is down-regulated, suggesting that YAP activation contributes to renal fibrosis. However, YAP activation does not always exert a detrimental effect and can also be beneficial. For instance, YAP activation in mice with ischemic injury mice facilitates renal functional and structural recovery, as evidenced by delayed recovery following treatment with verteporfin, a YAP inhibitor [37]. Additionally, we found that UUO in vivo or TGFβ in vitro could reduce p-YAP and enhance YAP expression. Interestingly, Zheng et al. [12] reported synchronous increases in YAP and p-YAP expression in lysophosphatidic acid-stimulated HK-2 cells and murine TECs. This discrepancy in YAP and p-YAP expression may be attributed to differing upstream mechanisms. In our study, UUO or TGFβ induced the up-regulation of EZH2 and H3K27me3, inhibited LATS1 and p-LATS1 expression, leading to reduced p-YAP levels and increased YAP nuclear translocation. This suggests that inhibiting YAP degradation may facilitate its nuclear transformation, resulting in opposing expression of p-YAP and YAP. Conversely, enhancing YAP transcription and translation could be simultaneously up-regulated, leading to their concurrent up-regulation.

The connection between the upstream regulators of YAP and renal fibrosis has been widely investigated. In 2020, researchers developed a double-knockout mouse model for MST1/2, key upstream regulators of the Hippo signaling pathway, and demonstrated that deletion of tubular MST1/2 increases YAP expression, thereby promoting renal fibrosis and functional impairment [9]. Significant progresses have been made in understanding the upstream mechanisms of the Hippo-YAP signaling pathway, yet the specific role of LATS1 in renal fibrosis remains unknown. LATS1, a crucial tumor suppressor within the Hippo signaling pathway, has recently been recognized as a novel actin-binding protein and a regulator of the cell-cycle [38]. In our study of UUO-induced renal fibrosis, inhibiting EZH2 with ZLD1039 restored LATS1 expression at both 7 and 14 days, indicating that LATS1 plays a protective role in renal fibrosis. Regarding the regulatory mechanism of LATS1, it has been shown that EZH2 and H3K27me3 can bind to the LATS1 promoter, thereby repressing LATS1 expression [39]. In renal cell carcinoma, tazemetostat treatment reduces H3K27me3 level and increases LATS1 expression, thereby exerting beneficial function [22]. Importantly, we propose that EZH2 and H3K27me3 inhibit LATS1 expression and activation, leading to reduced p-YAP levels and promoting YAP nuclear translocation, thereby regulating renal fibrosis. Therefore, in this study, we focused solely on the total LATS1 protein level and p-LATS1 level, rather than non-phosphorylated LATS1. Moreover, Co-IP evidence has revealed an interaction between EZH2 and YAP, suggesting a colocalization of these proteins. This finding provides important insights for further exploring the underlying molecular mechanisms. In summary, the initial EZH2-mediated reduction in LATS1 likely contributes, at least in part, to YAP activation in UUO-induced renal fibrosis.

Collectively, our findings demonstrate that ZLD1039 has the potential to ameliorate UUO-induced renal fibrosis and renal inflammation. In addition, our detailed mechanistic investigation expands the current knowledge about the interactions between epigenetics and the Hippo signaling pathway. Mechanically, our study identifies ZLD1039 as a potential anti-fibrotic therapeutic agent that attenuates renal fibrosis by inhibiting EZH2/H3K27me3 and up-regulating LATS1, thereby preventing YAP nuclear translocation (Fig. 7f). However, the direct relationship between EZH2/H3K27me3 and LATS1 remains to be fully elucidated, and the potential involvement of other molecular mediators linking EZH2 and YAP activation requires further investigation. Up to now, EZH2 has been implicated in kidney injury and renal fibrosis via multiple pathways. ZLD1039, a highly effective and orally bioavailable EZH2 inhibitor, has been preliminarily confirmed as safe in animals. Nevertheless, it must undergo multi-phase clinical trials and formulation studies before clinic application. Our ultimate goal is to facilitate its translational application and contribute to the development of novel therapeutic agents. Therefore, using the UUO model to simulate the renal fibrosis mechanism in human body and explore the effect and specific mechanisms of ZLD1039 on renal fibrosis could provide a robust basis for its future clinical transformation.

## Materials and methods

### Chemicals, antibodies and primer sequences

ZLD1039 kindly donated by the State Key of Laboratory of Biotherapy, Sichuan University (Sichuan, China). TGF-β was purchased from GenScript (Z03411, Genscript, China).

### Animal experiments

To achieve greater stability and reproducibility in our results, thereby minimizing variability introduced by hormonal changes, we use male rats in our study. Male Sprague − Dawley rats (6 − 8 weeks old, weight 180 − 220 g) were obtained from the Experimental Animal Center of Southwest Medical University. All animal experiment protocols were approved by the Ethics Committees of the Affiliated Hospital of Southwest Medical University and were in accordance with the standards of the Declaration of Helsinki. Twenty-four rats were randomly assigned to three groups: sham group (*n* = 8), UUO group (*n* = 8), UUO + ZLD1039 group (*n* = 8). After being anaesthetized with sodium pentobarbital (50 mg/kg), the rats in the UUO and UUO + ZLD1039 groups underwent left ureter ligation, while the sham group had their left ureter separated without ligation. Starting from the second day post-surgery, rats in the UUO + ZLD1039 group received daily gavage of 120 mg/kg ZLD1039, which was dissolved in 0.9% saline, and daily volume is about 1 ml/100 g. The sham and UUO groups received an equivalent volume of 0.9% saline. Each rat was fasted for at least 6 h before gavage administration. After 7 and 14 consecutive days of gavage administration, kidney tissues were collected for histological examination, qRT-PCR and Western Blot analysis.

### Cell culture

Rat renal tubular epithelial cells (NRK-52E) were purchased from the Chinese Tissue Culture Collections (CTCC-400–0381-CM, MeisenCTCC, China). All cell lines used in this study have undergone authentication to confirm their identity and tested mycoplasma free. The cells were cultured in DMEM containing 10% fetal bovine serum (FBS) and 1% penicillin and streptomycin, in an atmosphere of 5% CO_2_ at 37℃.

### Separation of nucleus and cytoplasm

NRK-52E cells were plated in 100 mm plates. After a 6-h starvation period, varying concentrations of ZLD1039 (0.4 and 0.8 µM) were added in the culture medium with or without TGF-β (10 µg/mL) and incubated for 72 h. Follow these steps to separate the nucleus and cytoplasm: (1) Discard the medium, add pre-cooled PBS to clean the cells and gently scrape off the cells with cell scraping, and then transfer the cell suspension to the EP tube; Centrifuge at 1000 rpm at 4℃ for 5 min; Cell precipitation was collected; (2) The lysis buffer containing protease inhibitor was added to the cell precipitation, blown and mixed repeatedly, and lysis was performed on a runner in a refrigerator at 4℃ for 20 min; (3) Add 10μL 10% NP-40, violently shake for 15 s, and leave it on ice for 2 min; Centrifuge at 12000 rpm at 4℃ for 15 min; The supernatant was cytoplasmic and denatured at 99℃ for 10 min after adding 5 × SDS. (4) Add 1 mL PBS to the precipitation, and vortex 15 s on the vortex meter, so that the precipitation is completely dispersed; Centrifuge at 12000 rpm at 4℃ for 5 min, discard the supernatant, and repeat the operation three times; The obtained precipitate is the nucleus. Add 100μL 1 × SDS loading buffer to the precipitate, blow and mix it repeatedly, add it at 99℃ for 10 min, and the obtained sample can be used for Western blot experiment.

### H&E and Masson’s trichrome staining

Kidney tissues were fixed in 4% paraformaldehyde and embedded with paraffin. The paraffin-embedded samples were sectioned at 4 μm thickness to perform Hematoxylin–Eosin (H&E) (C0105S, Beyotime, China) and Masson’s trichrome staining (BA4079B, BASO, China) to evaluate renal tubular injury and renal fibrosis. Cellular swelling or vacuolization, tubular dilation, loss of brush border, cast formation, and tubular necrosis and detachment were used to semi-quantitatively the extent of damage in renal tubules. Renal fibrosis was observed under an optical microscope (Nikon, Japan), and the area of renal fibrosis was measured using Image Pro-Plus Software.

### Immunohistochemistry (IHC)

Paraffin-embedded kidney tissues were sectioned to a thickness of 4 µm. The sections underwent a series of preparatory steps: deparaffinization, rehydration, antigen retrieval, endogenous peroxidase activity inhibition, and blocking of non-specific antigen-binding sites. Then, the tissue sections were incubated with the primary antibodies (Table 2) at 4℃ overnight. Subsequently, the sections were exposed to secondary antibodies and then stained, using 3,3'-diaminobenzidine (DAB) to enhance visualization of the reaction. Pictures were obtained using an optical microscope. Image Pro-Plus Software were used to count the mean optical density of the target proteins.

### Cell counting kit-8 (CCK-8) assay

The cell viability was assessed by the CCK-8 (CK04, Donjindo, Japan). NRK-52E cells were plated in 96-well plates. After a 6-h starvation period, varying concentrations of ZLD1039 (0.1 to 6.4 µM) were added in the culture medium with or without TGF-β (10 µg/mL) and incubated for 72 h. Subsequently, 10 µL of CCK-8 solution was added to 100 µL of culture medium and incubated for 1.5 h at 37℃. The absorbance at 450 nm was measured using a microplate reader to determine cell viability.

### Quantitative real time PCR (qRT-PCR)

Total RNA of kidney tissues or cells were extracted using the RNA isolater Total RNA Extraction Reagent (R401-01, Vazyme, China) according to the manufacturer’ s instructions. The purity and concentration of the RNA were measured on a NanoDrop2000 spectrophotometer. A 2-μg aliquot of RNA was reverse transcribed into cDNA using HiScript® III RT SuperMix for qPCR (+ gDNA wiper) (R323-01, Vazyme, China). Subsequently, quantitative PCR (qPCR) was conducted using ChamQ Universal SYBR qPCR Master Mix kit (Q711-02, Vazyme, China). The primer sequences are detailed in Table 1. mRNA levels were quantified by the 2^−ΔΔCq^ method and normalized to the endogenous reference gene GAPDH.

**Table 1:** The primers used in this study

Primer	Source	Forward (5’ - 3’)	Reverse (5’ - 3’)
GAPDH	rat	CGACTTCAACAGCAACTCCCACTCTTCC	TGGGTGGTCCAGGGTTTCTTACTCCTT
TNF-α	rat	CGGGCTCAGAATTTCCAACA	CGCAATCCAGGCCACTACTT
IL-6	rat	ACAAGTCCGGAGAGGAGACT	TTCTGACAGTGCATCATCGC
IL-1β	rat	ATCTCACAGCAGCATCTCGACAAG	CACACTAGCAGGTCGTCATCATCC
α-SMA	rat	GCGTGGCTATTCCTTCGTGACTAC	CCATCAGGCAGTTCGTAGCTCTTC
FN	rat	GGATCCCCTCCCAGAGAAGT	GGGTGTGGAAGGGTAACCAG
EZH2	rat	AGACCAGGCCCGAGTAT	GCAGTTGGAGTGTGCCG
LATS1	rat	TCCCGAATCCCGAGCGTT	GCTTCCTTCTGGGCCAATGT

### Co-immunoprecipitation (Co-IP)

To study the interaction between YAP and EZH2, cells were lysed in ice-cold RIPA (BL504 A, Biosharp, China) lysis buffer containing fresh protease inhibitors (P1081, Beyotime, China) and 1% PMSF (ST506, Beyotime, China) for 2 h. Then cell lysates were incubated with YAP or EZH2 antibody in conjunction with Protein G affinity beads (G2207, Servicebio, China) for 4 h at 4 °C. The binding complexes were washed twice and mixed with loading buffer for Western Blotting analysis.

### Western blotting analysis

Renal tissues or NRK-52E cells were lysed with RIPA lysis buffer containing phosphatase inhibitor cocktail and 1% PMSF for protein extraction. The protein concentration was measured using a BCA kit (P0012, Beyotime, China), and dissolved in 5 × SDS-PAGE sample loading buffer. Samples were separated by 8%−12% SDS-PAGE gels according to the different protein molecular weight and electro-transferred to nitrocellulose membranes (10600002, Cytiva, Germany). Followed by blocking with 5% non-fat milk, nitrocellulose membranes were incubated with corresponding primary antibody overnight on the shaker at 4 °C. Subsequently, the membranes were incubated with HRP-labeled Goat Anti-Rabbit IgG(H + L) (A0208, Beyotime, China) or HRP-labeled Goat Anti-Mouse IgG(H + L) (A0216, Beyotime, China) for 1 h at room temperature. Protein bands were visualized using ECL (P0018FS, Beyotime, China). Antibodies information used in the experiments is presented in Table 2.

**Table 2:** Antibodies information used in the experiments

Antibody	Source	Dilution	Cat No.	Application
α-SMA	HUABIO	1:2000, 1:5000	ET1607-53	IHC, WB
FN	Boster Biological Technology	1:250, 1:1000	BA1772	IHC, WB
EZH2	Cell Signaling Technology	1:50, 1:1000	5246	IHC, WB
IL-1β	ProteinTech Group	1:100, 1:1000	26048-1-AP	IHC, WB
IL-6	HUABIO	1:200, 1:1000	R1412-2	IHC, WB
TNF-α	ProteinTech Group	1:250, 1:1000	26405-1-AP	IHC, WB
H3K27me3	Cell Signaling Technology	1:200, 1:1000	9733	IHC, WB
Histone 3	Cell Signaling Technology	1:2000	4499	WB
p-LATS1	ProteinTech Group	1:5000	28998-1-AP	WB
LATS1	ProteinTech Group	1:1000	17049-1-AP	IHC, WB
TEAD1	Affinity Biosciences	1:1000	DF3141	WB
β-Actin	ABclonal Biotech	1:2000	AC004	WB
YAP	Cell Signaling Technology	1:1000	14074	WB
p-YAP	Cell Signaling Technology	1:1000	13008	WB
anti-Rat IgG	HUABIO	1:100000	HA1023	WB

### Statistical analysis

Data were presented as the mean ± SD. The differences between variables were compared by t-test or one-way analysis of variance (ANOVA). All statistical analyses were performed by GraphPad Prism 9.1. The statistical significance was set to *P* < 0.05.

## Supplementary Information

Supplementary Material 1.

## Data Availability

All the data are available from the corresponding author Li Wen (wenlixnydfy@163.com) upon reasonable request.
