# Supplementary material for: The EZH2 selective inhibitor ZLD1039 attenuates UUO-induced renal fibrosis by suppressing YAP activation
Source: Mol Biomed. 2025 Jun 6;6:36. doi: 10.1186/s43556-025-00276-5 (PMC12144025; doi:10.1186/s43556-025-00276-5)
Supplement: Supplementary file 1 — Supplementary Material 1. [file 43556_2025_276_MOESM1_ESM.docx]

**The EZH2 Selective Inhibitor ZLD1039 Attenuates UUO-Induced Renal Fibrosis by Suppressing YAP Activation**

Qingling Xia ^1, §^, Fujiang Xu ^2, §^, Lidan Zhang ^3^, Wenfei Ding ^1^, Jiang Liu ^1^, Jing Liu ^4^, Minhao Chen ^5^, Santao Ou ^1, *^, Yong Xu ^6, *^, Li Wen ^1, *^

^1^ Department of Nephrology, The Affiliated Hospital of Southwest Medical University; Sichuan Clinical Research Center for Nephropathy and Metabolic Vascular Diseases Key Laboratory of Sichuan Province, Luzhou, China.

^2^ Department of Oncology, The Affiliated Hospital of Southwest Medical University, Luzhou, China.

^3^ Laboratory of Anesthesia & Critical Care Medicine, Translational Neuroscience Center, West China Hospital of Sichuan University, Chengdu, China.

^4^ Department of Urology, The Affiliated Hospital of Southwest Medical University, Luzhou, China.

^5^ Clinical Medical College, Southwest Medical University, Luzhou, China.

^6^ Department of Endocrinology and Metabolism, The Affiliated Hospital of Southwest

Medical University, Luzhou, China.

^§^ These authors contributed equally to this work.

* Correspondence: Santao Ou, Yong Xu and Li Wen, The Affiliated Hospital of Southwest Medical University, No.25 Taiping Street, Jiangyang district, Luzhou, 646000, China. Tel.: +86-0830-3165341. E-mail: [ousantao@163.com(ST](mailto:ousantao@163.com(ST) Ou), xywyll@swmu.edu.cn (Y Xu), and wenlixnydfy@163.com (L Wen).


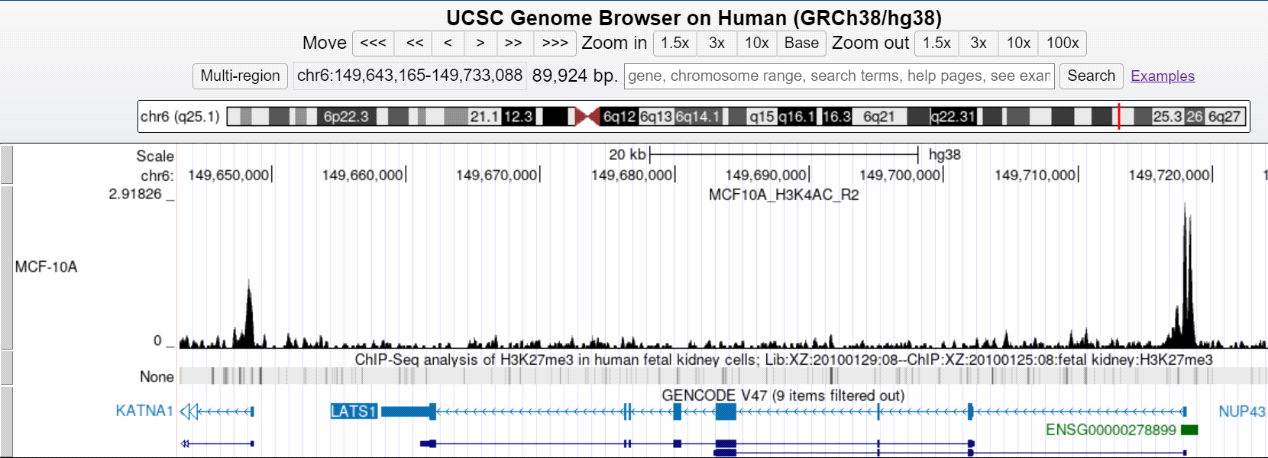


Fig.S1 The ChIP-seq results from the Cistrome Data Browser show a distinct enrichment peak of H3K27me3 in the promoter region of LATS1.
